# Psychological Morbidity in Obstructive Sleep Apnoea: Prevalence and Associated Factors in an Omani Cohort

**DOI:** 10.1192/bjo.2026.11197

**Published:** 2026-06-30

**Authors:** Salim Al-Huseini, Mohammed Qutishat, Said Al Kaabi, Mohammed Albalushi, Badar Al-Habsi

**Affiliations:** 1 Al Masarra Hospital, Ministry of Health, Muscat, Oman; 2 College of Nursing, Sultan Qaboos University, Muscat, Oman; 3 Al Masarra Hospital, Muscat, Oman

## Abstract

**Aims:**

Obstructive sleep apnoea (OSA) is a common sleep disorder linked to significant psychological issues, particularly depression and anxiety. The relationship between OSA and these mental health conditions is complex and has not been thoroughly studied in the Omani population.

This study aimed to assess the prevalence of depression and anxiety in patients diagnosed with OSA at Almasarra Hospital (AMH) in Oman and to identify related socio-demographic and clinical factors.

**Methods:**

This cross-sectional study involved 108 patients diagnosed with OSA, with data collected during routine follow-ups at AMH. Structured questionnaires were used to gather demographic information and responses to the Hospital Anxiety and Depression Scale (HADS). Statistical analyses included descriptive statistics, Pearson correlation, and regression models to identify predictors of depression and anxiety.

**Results:**

The study found that 22.2% of participants exhibited depressive symptoms, while 17.6% showed anxiety symptoms. A significant positive correlation was found between anxiety and depression scores (r=0.680, p<0.01). Regression analysis revealed that higher BMI and employment status were significant predictors of both depression and anxiety (p<0.05). The mean age of participants was 38.35 years, with a notable prevalence of overweight/obesity (mean BMI=32.31). While sleep severity indices showed only marginal associations, psychosocial factors appeared to have a greater impact.

**Conclusion:**

A significant proportion of Omani patients with OSA experience symptoms of depression and anxiety, which are interconnected and influenced by lifestyle factors. Incorporating mental health assessments into routine OSA management may enhance patient outcomes and overall quality of life.

